# Enhanced Photocatalytic Hydrogen Evolution by Loading Cd_0.5_Zn_0.5_S QDs onto Ni_2_P Porous Nanosheets

**DOI:** 10.1186/s11671-018-2438-0

**Published:** 2018-02-02

**Authors:** Lingfeng Xiao, Tong Su, Zhuo Wang, Kun Zhang, Xiaoniu Peng, Yibo Han, Quan Li, Xina Wang

**Affiliations:** 1 Hubei Collaborative Innovation Center for Advanced Organic Chemical Materials, Hubei Key Laboratory of Ferro & Piezoelectric Materials and Devices, Faculty of Physics and Electronic Science, Hubei University, Wuhan, 430062 China; 2 State Center for Designer Low-Carbon and Environmental Materials, Zhengzhou University, 100 Kexue Avenue, Zhengzhou, 450001 China; 3 Wuhan National High Magnetic Field Center and School of Physics, Huazhong University of Science and Technology, Wuhan, 430072 China; 4 Department of Physics, The Chinese University of Hong Kong, Hong Kong, China

**Keywords:** Ni_2_P, Cd_0.5_Zn_0.5_S, Nanosheet, Quantum dot, Hydrogen evolution

## Abstract

Ni_2_P has been decorated on CdS nanowires or nanorods for efficient photocatalytic H_2_ production, whereas the specific surface area remains limited because of the large size. Here, the composites of Cd_0.5_Zn_0.5_S quantum dots (QDs) on thin Ni_2_P porous nanosheets with high specific surface area were constructed for noble metal-free photocatalytic H_2_ generation. The porous Ni_2_P nanosheets, which were formed by the interconnection of 15–30 nm-sized Ni_2_P nanoparticles, allowed the uniform loading of 7 nm-sized Cd_0.5_Zn_0.5_S QDs and the loading density being controllable. By tuning the content of Ni_2_P, H_2_ generation rates of 43.3 μM h^− 1^ (1 mg photocatalyst) and 700 μM h^− 1^ (100 mg photocatalyst) and a solar to hydrogen efficiency of 1.5% were achieved for the Ni_2_P-Cd_0.5_Zn_0.5_S composites. The effect of Ni_2_P content on the light absorption, photoluminescence, and electrochemical property of the composite was systematically studied. Together with the band structure calculation based on density functional theory, the promotion of Ni_2_P in charge transfer and HER activity together with the shading effect on light absorption were revealed. Such a strategy can be applied to other photocatalysts toward efficient solar hydrogen generation.

## Background

As an efficient strategy to produce H_2_ by utilizing solar energy, photocatalytic hydrogen production has attracted extensive attention since TiO_2_ was reported as a photocatalyst in 1972 [1]. Compared with TiO_2_, Cd_x_Zn_1−x_S shows excellent visible-light driven catalytic activity because of the narrower band gap and good photochemical stability. A H_2_ production rate as high as 1097 μM h^− 1^ g^− 1^ has been achieved by using Cd_0.5_Zn_0.5_S as photocatalyst [2], which composition has been proven to be the optimum for photocatalytic property. To decrease carrier recombination and prompt carrier separation for hydrogen evolution reaction (HER), noble metals such as Pt, Co-Pt, Ru, Au, and Pd have been used as co-catalysts [3–8]. For example, when co-catalyzed with Co-Pt, the photocatalytic H_2_ generation rate of Cd_0.5_Zn_0.5_S quantum dots (QDs) could be increased by 4.7-folds [4]. A H_2_ production as high as ~ 6.3 mM h^− 1^ mg^− 1^ was achieved when CdZnS was combined with Au [9]. However, the high cost of noble-metals greatly limits the future application in large scale, which makes the non-precious co-catalysts to be good candidates of precious ones for photocatalytic H_2_ generation.

Among the various non-noble co-catalysts including carbon family (graphene, carbon nanotubes, reduced grapheme oxide, carbon nanodots) [10–15], phosphides [16–22], and TiO_2_ [23, 24] and sulfides [25–32], Ni_2_P and CoP have been extensively composited with CdS nanowires and/or nanorods for efficient photocatalytic H_2_ production [16–18, 33–36]. In these composites, one-dimensional (1D) CdS was always decorated by smaller phosphides’ nanoparticles or nanosheets with HER activity, and carrier recombinations can be greatly reduced because of the long carrier diffusion length of the 1D structure and its well-defined hetero-interface with the co-catalysts. Considering the advantages of QDs such as its high solar energy to fuel conversion efficiency, low fabrication costs [37, 38], and HER mainly occurs at co-catalyst/electrolyte interface, it is rational to construct hetero-nanostructures with plenty of specific surface area of active sites while still maintaining fast carrier separation. In this case, a reverse structure with photocatalysts loaded onto co-catalysts was reported for efficient photocatalytic H_2_ generation [10, 13]. For instance, hydrogen generation rates of 2.08 and ~ 33.4 mM h^− 1^ mg^− 1^ were established by loading Cd_0.5_Zn_0.5_S QDs onto onion-like carbon and 2D graphitic carbon nitride (g-C_3_N_4_) microribbons, respectively. These make it highly expectable for photocatalytic H_2_ generation if phosphide nanostructures were decorated by Cd_0.5_Zn_0.5_S QDs. However, such a reverse structure has been rarely reported up to now.

Here, a reverse structure of Cd_0.5_Zn_0.5_S QDs on Ni_2_P nanosheet arrays was synthesized by thermal solution method for enhanced photocatalytic H_2_ generation. A hydrogen generation rate of 700 μM h^− 1^ (with 100 mg feeding catalyst) and a solar to hydrogen efficiency (STH) of 1.5% were achieved at 1.5 wt% of Ni_2_P. The effect of Ni_2_P on the H_2_ generation rate, optical, and electrochemical property of the composite was systematically studied. Moreover, the band structure of Ni_2_P was calculated based on density functional theory, together with the photo-electrochemical property, the detailed role of Ni_2_P for the H_2_ generation was revealed.

## Methods/experimental

### Synthesis of Co-catalyst

Firstly, 20 mL deionized water containing 2.61 g nickel nitrate and 2.52 g hexamethylenetetramine was transferred to a Teflon autoclave and heated at 120 °C for 10 h for the formation of NiOOH. After cooled down to room temperature, the NiOOH product was washed by alcohol and deionized water via centrifugation at 2000 rpm for three times and each time for 5 min. Then, a mixture of 0.22 g NiOOH and 0.44 g sodium hypophosphite was put into a tube furnace and heated at 500 °C for 2 h for phosphorizing. When it naturally cooled down to room temperature, black Ni_2_P powder was obtained and collected.

### Synthesis of Ni_2_P-Cd_0.5_Zn_0.5_S Nanocomposites

To prepare Ni_2_P-Cd_0.5_Zn_0.5_S composite, 100 mg Ni_2_P powder was dispersed into 20 mL ethanol via ultrasonic processing for 1 h. Then x mL (*x* = 0.48, 0.96, 1.4, 3, 5) well-dispersed Ni_2_P solution was added into a 20 mL ethylene glycol solution containing 272.6 mg ZnCl_2_ and 456.7 mg CdCl_2_∙2.5H_2_O, and was heated to 170 °C with continuous stirring under nitrogen protection. After the addition of 20 mL ethylene glycol solution dissolving 960.7 mg Na_2_S∙9H_2_O, the solution was heated to 180 °C and held for 1 h for the growth of Cd_0.5_Zn_0.5_S on Ni_2_P. Finally, the samples were washed by alcohol and deionized water respectively for three times. By weighing the final xNi_2_P-Cd_0.5_Zn_0.5_S composites, the weight percents (wt%) were determined to be 0.5 (*x* = 0.48), 1 (*x* = 0.96), 1.5 (*x* = 1.4), 3 (*x* = 3), 5 (*x* = 5). As a comparison, pure Cd_0.5_Zn_0.5_S QDs were synthesized via the similar method except the addition of Ni_2_P.

### Morphology, Structure, and Optical Properties Characterization

The morphology, microstructure, and composition were characterized by field emission scanning electron microscopy (FESEM, JSM-7100F, JEOL) and transmission electron microscopy (TEM, FEI Tecnai 20) equipped with scanning transmission electron microscopy (STEM) and energy dispersive X-ray spectroscopy (EDX). Powder X-ray diffraction (XRD) patterns were recorded on a Bruker AXS D8 X-ray diffractometer with Cu Kα (λ = 1.54056 Å). Elemental composition, chemical, and valence states were studied by (valence band) X-ray photoelectron spectroscopy (XPS) measurements (XPS, Escalab 250Xi) with Al Kα radiation. UV-Vis absorption was investigated by an UV-Vis spectrophotometer (UV-3600, Shimadzu) equipped with an integrating sphere device, and the weight/volume ratio of sample to deionized water was kept at 1 mg/10 mL. Photoluminescence (PL) measurements were carried out on a 7000 FL spectrophotometer (Hitachi, F7000) with an excitation wavelength of 400 nm. Before the PL measurements, pure Cd_0.5_Zn_0.5_S QDs and the composites were well dispersed in ethanol, and the concentration of Cd_0.5_Zn_0.5_S was maintained at 0.5 mg/mL for all the samples.

### Linear Sweep Voltammetry (LSV) and Electrochemical Impedance Spectra (EIS) Measurements

LSV measurements were conducted in 1 M NaOH electrolyte (pH = 14) in an electrochemical work station (CHI 760E, CH Instruments) with a typical three-electrode configuration. A Pt foil and a saturated Ag/AgCl were used as the counter and reference electrode, respectively. The potentials were converted to those vs reversible hydrogen electrode (RHE) by the equation E(vs RHE) = E(vs Ag/AgCl) + E_Ag/AgCl_ (ref) + 0.0591 V × pH, where (E_Ag/AgCl_ (ref) = 0.1976 V vs NHE (normal hydrogen electrode) at 25 °C) [39]. Electrochemical impedance spectra (EIS) measurements were carried out in darkness at 0.5 V vs RHE with an amplitude of 5 mV and the electrolyte of 0.35 M Na_2_SO_3_ and 0.25 M Na_2_S aqueous solution by using a similar three-electrode system. The working electrode was made via spreading ~ 2 mg product (dispersed in 5 mL ethanol) over 4 cm^2^ area FTO substrate and dried at 70 °C for 5 h. The frequency range was kept within 0.1 Hz ~  100 kHz, and the spectra were analyzed by the Z-View program (Scribner Associates Inc.).

### Photocatalytic (PC) H_2_ Generation

Before H_2_ production, the photocatalysts with different mass (1, 5, and 10 mg) were dispersed in a sealed quartz reactor (volume 40 mL, 5 cm × 5 cm × 1.6 cm) with 15 mL 0.75 M Na_2_S and 1.05 M Na_2_SO_3_ aqueous solution. After degassing for 30 min by nitrogen, the photocatalytic experiment was performed under the irradiation of a 300 W Xe (PLS-SXE300/300UV, Perfect Light) lamp with a cut-off filter of 420 nm and an incident power of 300 mW/cm^2^. The catalytic solution was kept continually stirring during the whole PC experiment. In every hour, 1-mL gas production was collected and analyzed by a gas chromatograph (GC-2018, Shimadzu, Japan, TCD). Further cycling stability experiment was performed under the same condition. Paralleling experiments with the feeding dosage of photocalysts from 15 to 100 mg were conducted in 100 mL electrolyte of Na_2_S and Na_2_SO_3_ in a larger reactor (volume 150 mL) under the same illumination. The solar to hydrogen efficiency (STH) was calculated by the flowing equation:

$$ {\displaystyle \begin{array}{l}\mathrm{STH}\ \left(\%\right)=\kern0.5em \frac{\mathrm{energy}\ \mathrm{of}\ \mathrm{generated}\ {\mathrm{H}}_2}{\mathrm{light}\ \mathrm{energy}\ \mathrm{onto}\ \mathrm{the}\ \mathrm{surface}\ \mathrm{of}\ \mathrm{solution}}\times 100\%\\ {}\kern6.5em =\frac{237\mathrm{KJ}/\mathrm{mole}\kern0.5em \times \mathrm{moles}\ \mathrm{of}\ {\mathrm{H}}_2\ \mathrm{producted}}{\mathrm{area}\ \mathrm{of}\ \mathrm{solution}\ \mathrm{been}\ \mathrm{irradiated}\times 300\mathrm{mW}/{\mathrm{cm}}^2}\times 100\%\end{array}} $$

### Computational Methods

The energy and electronic properties of bulk Ni_2_P were calculated using density functional theory (DFT) method. Vienna Ab-initio Simulation Package (VASP) [40] was adopted during the calculations with the projector augmented wave pseudo potentials (PAW) [41], and the Perdew-Burke-Ernzerhof type (PBE) generalized gradient approximation (GGA) [42] exchange–correlational functional methods. A Brillouin zone with a 9 × 9 × 9 Monkhorst−Pack Γpoint grid [43], a kinetic energy cut off with 450 eV, and an energy criterion of 10^− 6^ eV were applied for geometric optimization until the residual forces were converged to less than 0.01 eV/Å. The bulk model of hexagonal Ni_2_P with P-62M symmetry was taken into account. After fully structure optimized, the lattice parameter of Ni_2_P (*a* = *b* = 5.86918 Å, and *c* = 3.37027 Å) can be obtained, which is well consistent with the reported values [44].

## Results and Discussion

**Fig. 1:**
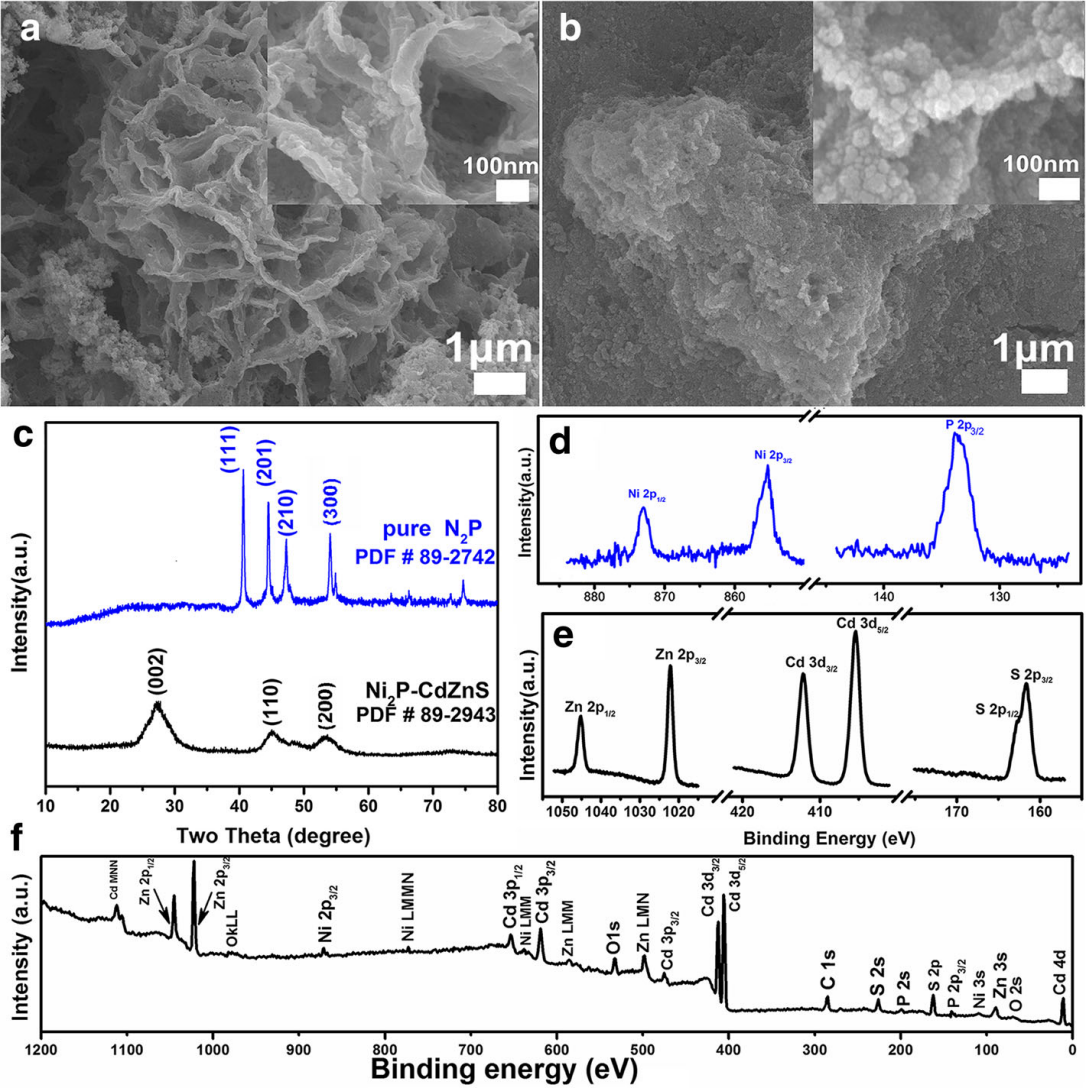
Morphology, crystalline property, and chemical states of Ni_2_P-Cd_0.5_Zn_0.5_S composites (1.5 wt% Ni_2_P). **a–b** Low and high (inset) magnification SEM images of Ni_2_P before and after the loading of Cd_0.5_Zn_0.5_S, **c** XRD pattern of Ni_2_P and Ni_2_P-Cd_0.5_Zn_0.5_S, **d–f** XPS fine and survey spectra of Ni_2_P-Cd_0.5_Zn_0.5_S composite

Figure 1a, b show the morphology of Ni_2_P before and after the composition with Cd_0.5_Zn_0.5_S QDs (Ni_2_P wt%: 1.5%). Pure Ni_2_P has a flower-like morphology which is composed of many crossed nanosheets with the thickness less than 20 nm and planar size from several tens nanometer to micrometer scope. From the XRD pattern of pure Ni_2_P in Fig. 1c, diffraction peaks of (111), (201), (210), and (300) planes can be clearly observed at 40.7°, 44.6°, 47.4°, and 54.2°, respectively, which correspond to hexagonal Ni_2_P (JCPDF no. 89-2742). After loaded by Cd_0.5_Zn_0.5_S QDs, the surface of the nanosheets become rather rough, and plenty of nanoparticles with size less than 10 nm can be distinguished on the pristine Ni_2_P skeleton. At the same time, the XRD refraction peaks of Cd_0.5_Zn_0.5_S (JCPDF no. 89-2943) (100), (002), (101), and (110) planes can be clearly found at 26.0°, 27.8°, 29.6°, and 45.9°, respectively [6, 45], while the diffraction signal of Ni_2_P is greatly depressed because of the low weight ratio (1.5 wt%) of Ni_2_P to Cd_0.5_Zn_0.5_S. The coexistence of Cd_0.5_Zn_0.5_S and Ni_2_P was demonstrated by the X-ray photoelectron spectrometer (XPS) fine and survey spectra in Fig. 1d–f. Except the oxygen and carbon signals arising from the air absorption, only Ni, P, Cd, Zn, and S can be detected, which rules out the possibility of other impurities. The peaks at 855.5 and 873.9 eV can be assigned to Ni 2p_3/2_ and 2p_1/2_, respectively, and the peak of P 2p_3/2_ can be found at 133.6 eV [16, 46]. Concurrently, the doublet peaks of Zn 2p, Cd 3d, and S 2p suggest the bivalent Zn^2+^, Cd^2+^, and S^2−^ from Cd_0.5_Zn_0.5_S QDs [3, 34, 47]. In brief, the growth of Cd_0.5_Zn_0.5_S on Ni_2_P nanosheets has been established for the formation of Ni_2_P-Cd_0.5_Zn_0.5_S nanocomposites.

**Fig. 2:**
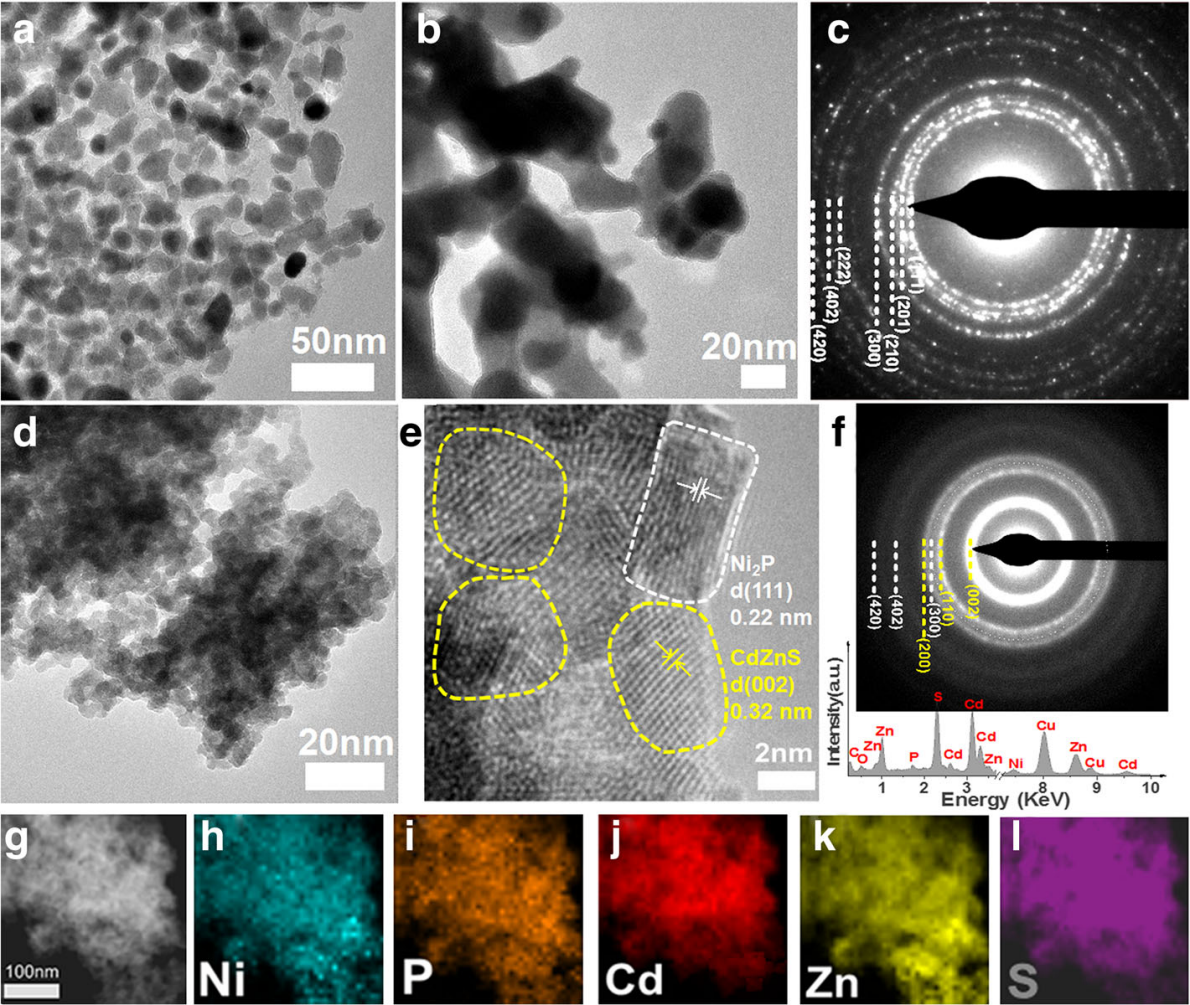
Microstructure of Ni_2_P and Ni_2_P-Cd_0.5_Zn_0.5_S composite. **a–c** and **d–f** Different-magnification TEM images and SAED pattern of Ni_2_P and Ni_2_P-Cd_0.5_Zn_0.5_S, the inset **f** is EDX spectrum, where the yellow and white dash lines denote Cd_0.5_Zn_0.5_S and Ni_2_P, respectively. **g** High-angle annular dark field (HAADF)-STEM image, and **h–l** the corresponding EDX mappings of Ni_2_P-Cd_0.5_Zn_0.5_S composite

The microstructure and elemental composition of the samples were further investigated by TEM-related techniques. From the different magnification TEM images of pure Ni_2_P (Fig. 2a, b), the nanosheets are porous and composed of cross-linked irregular nanoparticles with size of ~ 15–30 nm. The selected area electron diffraction pattern (SAED) in Fig. 2c shows the diffraction ring of Ni_2_P (111), (201), (210), and (300) planes. The diffractive signals of high-index planes such as (222), (402), and (420) can also be detected due to the strong multi-scattering of the high-energy electrons. After composited with Cd_0.5_Zn_0.5_S, the intercrossed Ni_2_P nanosheets were covered by plenty of smaller nanoparticles with size of ~ 7 nm (Fig. 2d). The EDX spectra (inset, Fig. 2f) clearly shows the signal of Ni, P, Cd, Zn, and S, indicative of the coexistence of Ni_2_P and Cd_0.5_Zn_0.5_S. From the SAED pattern (Fig. 2f), strong diffractive rings of Cd_0.5_Zn_0.5_S (002), (110), and (200) planes (denoted by yellow dash lines) can be clearly distinguished along with the weak signals of Ni_2_P (300), (402), and (420) (marked by white dash lines), suggesting the good composition of Ni_2_P with QDs. It is noticeable that Ni_2_P (300) ring overlaps with Cd_0.5_Zn_0.5_S (110) and (200) planes, making it hard to be distinguished. The high-resolution TEM image of Ni_2_P-Cd_0.5_Zn_0.5_S sample in Fig. 2e further shows the lattice fringes with spacing of 0.34 and 0.22 nm, which corresponds to the Cd_0.5_Zn_0.5_S (002) and Ni_2_P (111) crystal planes, respectively. The elemental EDX mappings (Fig. 2h–l) taken from the region shown by the high-angle annular dark field (HAADF) image (Fig. 2g) exhibit that Ni, P, Cd, Zn, and S are distributed uniformly among the sample, further demonstrating the successful composition of Cd_0.5_Zn_0.5_S QDs with the porous Ni_2_P nanosheets.

**Fig. 3:**
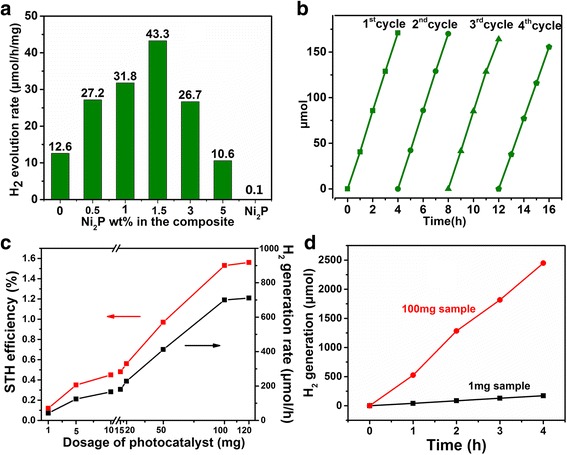
Photocatalytic property of Ni_2_P-Cd_0.5_Zn_0.5_S composites. **a** Photocatalytic hydrogen generation at different wt% of Ni_2_P and **b** the cycling test of the composite with 1.5 wt% of Ni_2_P tested in a small reactor (40 mL, 1.0 mg photocatalyst). **c** Hydrogen production rate and solar to hydrogen efficiency (STH) at various amount of photocatalyst. The tests for the photocatalyst of dosage from 15 to 100 mg were carried out in a 150 mL reactor, and those of dosage from 1 to 10 mg were in a 40 mL reactor. **d** The hydrogen generation rate for 1 and 100 mg composite samples (1.5 wt% Ni_2_P)

Figure 3a shows the H_2_ evolution rate of Ni_2_P-Cd_0.5_Zn_0.5_S nanocomposites varied with the content of Ni_2_P at the feeding dosage of 1 mg in a 40 mL reactor. Pure Cd_0.5_Zn_0.5_S shows a photocatalytic H_2_ evolution rate of 12.6 μM h^− 1^ mg^− 1^, and pure Ni_2_P shows negligible hydrogen generation. With the addition of Ni_2_P, the photocatalytic activity of the Ni_2_P-Cd_0.5_Zn_0.5_S composites has been obviously enhanced and reaches the highest value of 43.3 μM h^− 1^ mg^− 1^ at 1.5 wt% Ni_2_P, nearly 3.4 times higher than pure Cd_0.5_Zn_0.5_S. Further addition of Ni_2_P (≥ 3 wt%) will result in fast degradation of property, and the H_2_ evolution rate is less than pure Cd_0.5_Zn_0.5_S when Ni_2_P increases to 5 wt%. Such a non-linear behavior suggests that there exist an optimum Ni_2_P content, namely, an appropriate loading density of Cd_0.5_Zn_0.5_S on Ni_2_P for the photocatalytic property. At the same time, the stability of 1.5 wt% Ni_2_P-Cd_0.5_Zn_0.5_S was studied by cycling test (Fig. 3b). During four successive cycles that lasted for totally 16 h, the H_2_ generation maintained relative stable with negligible degradation, indicating the good photocatalytic stability of the composite.

The effect of the amount of catalyst on STH efficiency and H_2_ generation was systematically studied (Fig. 3c–d) for 1.5 wt% Ni_2_P-Cd_0.5_Zn_0.5_S sample. Two typical reactors with volume of 40 and 150 mL were adopted at the same illumination power density. When tested in the smaller reactor (40 mL), though both the STH and H_2_ generation rate increase with the catalyst’s dosage from 1 to 10 mg, the increased step is far less than that of the dosage. The STH and H_2_ generation rate are only 0.45% and 166 μM h^− 1^ when the dosage of the catalyst increased to 10 mg, nearly 3.8 times of the 1 mg sample. For the larger reactor (150 mL), distinct increase in STH and H_2_ generation can be found with the dosage increased from 15 to 100 mg, and a 1.53% STH and a 700 μM h^− 1^ of H_2_ generation can be achieved at the dosage of 100 mg, nearly 3.1 times of the 15 mg catalyst. Considering that the incident light has longer path when it passes through a deeper reactor, such a result shows that larger reactor will be more beneficial for the utilization of the incident light. However, the STH efficiency will be saturated once the dosage increased to about 100 mg, suggesting there exists an optimum dosage for the light utilization. The optimum H_2_ generation rate is superior than CdZnS QDs-2D g-C_3_N_4_ microribbons (H_2_ generation rate 33.4 mM h^− 1^ g^− 1^) [10], Cd_0.1_Zn_0.9_S nanoparticles-carbon nanotubes (rate: 1563 μM h^− 1^ g^− 1^) [11], a sandwich-structured C_3_N_4_/Au/CdZnS photocatalyst (rate 6.15 mM h^− 1^ g^− 1^) [9], and CdS QDs-sensitized Zn_1−x_Cd_x_S solid solutions (rate 2128 μM h^− 1^ g^− 1^) [48].

**Fig. 4:**
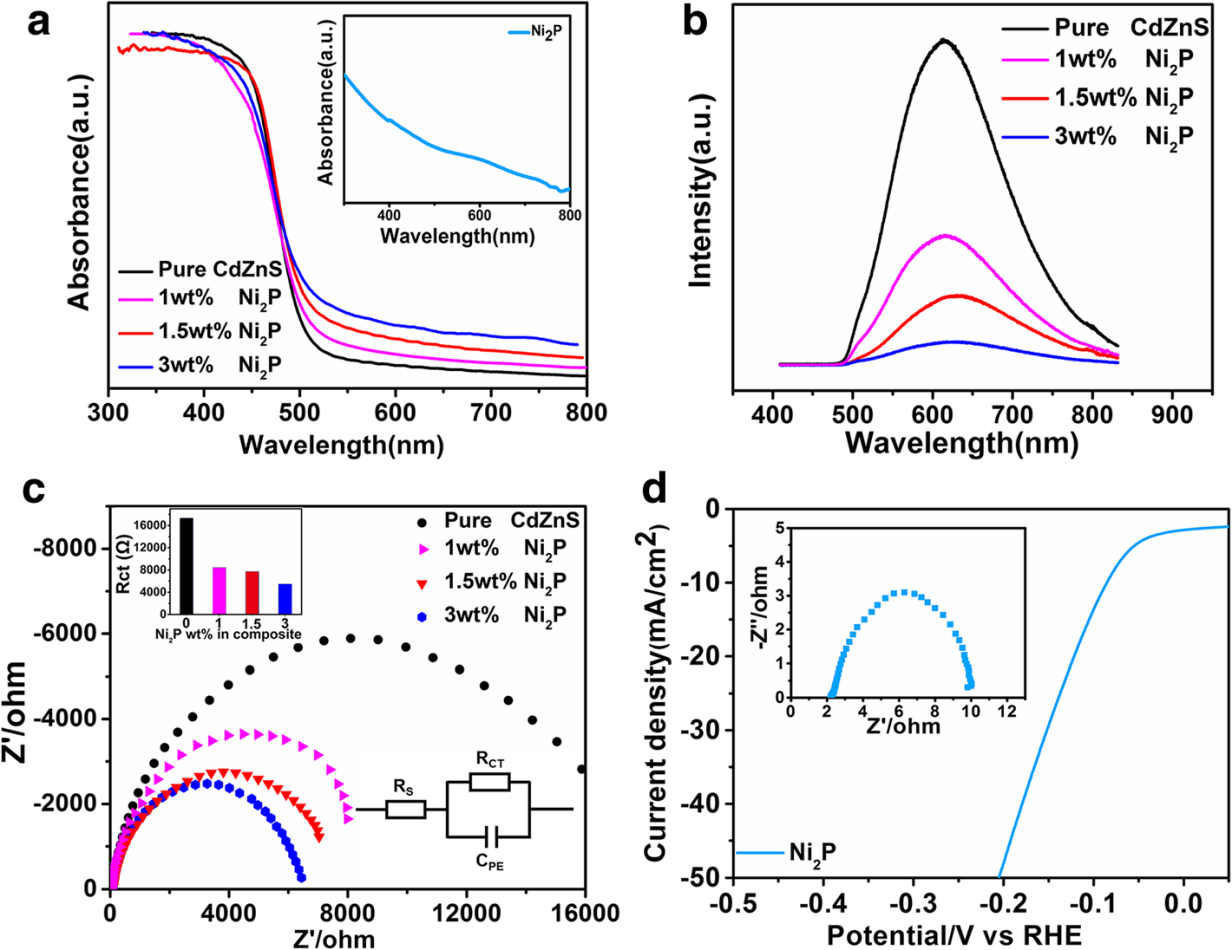
The effect of Ni_2_P content on the optical and electrochemical properties of Ni_2_P-Cd_0.5_Zn_0.5_S composite. **a** UV-Vis absorption spectra (inset pure Ni_2_P), **b** photoluminescence spectra, and **c** EIS spectra. **d** LSV curve and EIS (inset) spectrum of pure Ni_2_P

To reveal the mechanism for the enhanced photocatalytic property and detailed role of Ni_2_P, both the optical and electrochemical property of pure Ni_2_P, Cd_0.5_Zn_0.5_S, and the composites were studied by Fig. 4. From the absorption spectra (Fig. 4a), pure Cd_0.5_Zn_0.5_S exhibits an absorption edge at 506 nm, corresponding to the band gap of 2.45 eV [13, 49]. For pure Ni_2_P (the inset), wide absorption over the whole visible range can be found. After the composition, besides the absorption in range < 506 nm, obvious tails over the visible wavelength > 506 nm can be found, which can be attributed to the contribution from Ni_2_P. As the visible absorption in longer wavelength increases with Ni_2_P, the composite shows reduced absorption of Cd_0.5_Zn_0.5_S (< 506 nm). At the same time, the photoluminescence spectra (Fig. 4b) exhibit that pure Cd_0.5_Zn_0.5_S has intensive band edge luminescence at ~ 620 nm when excited at the wavelength of 400 nm. After composition, it will be degraded gradually with the addition of Ni_2_P. Considering that higher content of Ni_2_P will induce more Ni_2_P/Cd_0.5_Zn_0.5_S interfaces which help to enhance charge transfer and suppress charge recombination, the decrease of PL intensity can be understood by the reduced carrier recombination and enhanced charge transfer at the Ni_2_P/Cd_0.5_Zn_0.5_S interface.

The effective role of Ni_2_P in prompting charge transfer can also be reflected by the EIS spectra depending on Ni_2_P content (Fig. 4c). As shown by the equivalent circuit (inset, Fig. 4c), the charge transfer resistance (Rct) at catalyst/electrolyte interface can be evaluated by the semicircle radius of the Nyquist plots based on R-C equivalent circuit. The equivalent series resistance (ESR) can be obtained from the intersection of the curve and the real resistance (*Z*’) axis, while the charge-transfer resistance (Rct) corresponds to the width of the semicircle plotted at higher frequencies. The R_CT_ of pure Cd_0.5_Zn_0.5_S is 17,320 Ω, indicative of its semiconductor nature. After the composition with 1, 1.5, and 3 wt% Ni_2_P, R_CT_ decreases gradually to 8432, 7721, and 5473 Ω, respectively, suggesting the enhancement of Ni_2_P in the electrical conductivity. Indeed, Ni_2_P has been considered as a good electrocatalyst toward HER [44, 50, 51]. From the LSV curve of pure Ni_2_P on Ni foam shown in Fig. 4d, the Ni_2_P has good HER activity with overpotentials of 84 mV and 201 mV to attach the current density of 10 and 50 mA/cm^2^ (without iR-correction), respectively. The EIS spectrum (inset Fig. 4d) exhibits that Ni_2_P has a very low R_CT_ (~ 7.3 Ω), indicating the metallic character of Ni_2_P. Therefore, Ni_2_P can not only increase the electrical conductivity at Cd_0.5_Zn_0.5_S/Ni_2_P interface, but also supply effective active sites for HER, then leading to enhanced photocatalytic property of the composite.

**Fig. 5:**
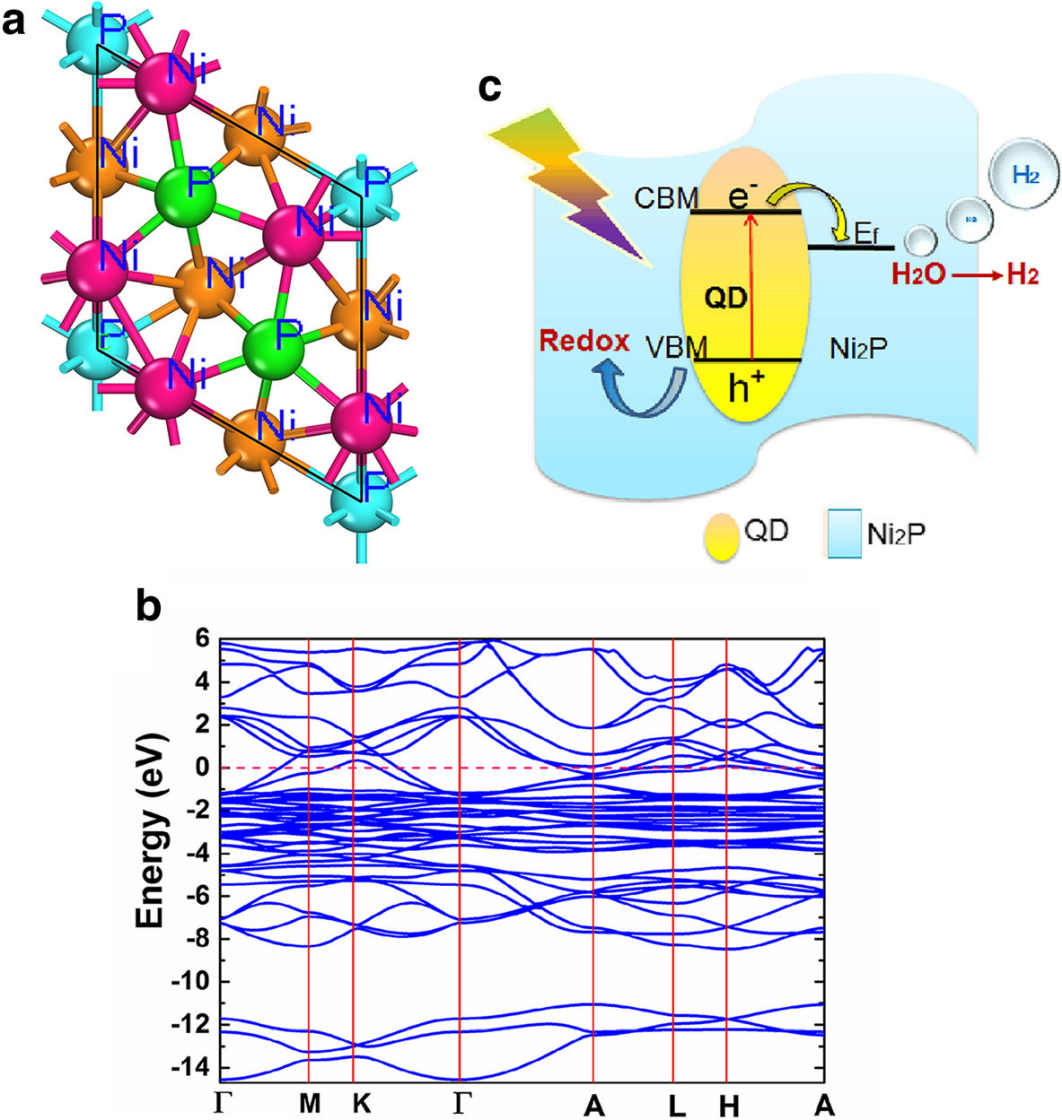
The band diagram and charge separation and transfer mechanism for the photocatalytic H_2_ evolution. **a** Top views of the ball and stick model of (001) surface-terminated bulk Ni_2_P. **b** Calculated band structure of Ni_2_P where the red dash line represents Fermi level. **c** Schematic mechanism illustrating the charge separation and transfer for the photocatalytic H_2_ generation

Considering that the addition of Ni_2_P decreased the absorption at wavelength < 506 nm, it is necessary to demonstrate whether the light absorption of Ni_2_P can be utilized to generate hydrogen. The band structure of Ni_2_P was then studied by DFT calculation. Figure 5a, b presents the ball and stick model of bulk Ni_2_P and the calculated band structure. From Fig. 5b, no band gap can be detected, suggesting the metallic characteristic of Ni_2_P, which agrees well with the above EIS result. This indicates that the photoelectrons are mainly attributed to the photo-excitation of Cd_0.5_Zn_0.5_S rather than Ni_2_P. Moreover, the Fermi level of Ni_2_P (obtained from out car file) locates at 1.03 V vs. NHE, much lower than the conductive band minimum (CBM) level (− 1.04 V vs NHE) of Cd_0.5_Zn_0.5_S QDs [13].

Accordingly, the schematic mechanism was demonstrated for the photocatalytic H_2_ evolution of the composite by Fig. 5c. The location of Fermi level of Ni_2_P makes it energetically favorable for the transfer of photo-generated electrons from Cd_0.5_Zn_0.5_S to Ni_2_P, then prompts the separation of photo-excited electrons and holes at the interface, resulting in the depression of charge recombination. Concurrently, H_2_ will evolve efficiently at the active sites of Ni_2_P due to the good HER activity and large specific surface area of the composites. The positive roles of Ni_2_P in charge transfer and HER activity will dominate at the lower content of Ni_2_P (≤ 1.5 wt%). When the content surpasses 1.5 wt%, the shading effect of Ni_2_P in light absorption will overcome the positive aspect, leading to the degradation of H_2_ generation. An optimum photocatalytic property will be achieved at 1.5 wt% Ni_2_P when the two effects reach a balance.

## Conclusions

A reverse structure of Cd_0.5_Zn_0.5_S QDs on Ni_2_P porous nanosheets were fabricated for efficient photocatalytic H_2_ production. The Ni_2_P porous nanosheets were composed of 15–30-nm-sized nanoparticles that allows the effective loading of 7-nm-sized Cd_0.5_Zn_0.5_S QDs. As the charge separation and transfer property is enhanced with the addition of Ni_2_P from 0 to 5 wt%, a competitive shading effect that unbeneficial for the light absorption of Cd_0.5_Zn_0.5_S is induced. An optimum photocatalytic H_2_ generation of 43.3 μM h^− 1^ (dosage 1 mg) will be achieved at 1.5 wt% Ni_2_P. Based on the optimum content, the photocatalytic dependence on feeding dosage of catalyst shows that the STH efficiency will reach the highest value of 1.5% at the dosage of 100 mg. The high HER activity and band structure of Ni_2_P were revealed, confirming the effective role of Ni_2_P in prompting photocatalytic H_2_ evolution dynamics from both experimental and theoretical aspects. The heterostructure of Cn_0.5_Zn_0.5_S QDs-Ni_2_P porous nanosheets can not only help to prompt the photo-excited charge separation and transfer, but also speed up the dynamics of hydrogen evolution reaction via the co-catalytic role of N_i2_P, thus enhances the photocatalytic hydrogen generation property. Such a method can be applied to other catalysts toward efficient photocatalytic property.

## Abbreviations

CBM: Conductive band minimum
DFT: Density functional theory
EDX: Energy dispersive X-ray spectroscopy
EIS: Electrochemical impedance spectra
FESEM: Field emission scanning electron microscopy
FTO: Fluorine-doped tin oxide
GGA: Generalized gradient approximation
HER: Hydrogen evolution reaction
LSV: Linear sweep voltammetry
NHE: Normal hydrogen electrode
PBE: Perdew-Burke-Ernzerhof type
PC: Photocatalytic
PL: Photoluminescence
QDs: Quantum dots
RHE: Reversible hydrogen electrode
STEM: Scanning transmission electron microscopy
STH: Solar to hydrogen
TEM: Transmission electron microscopy
VASP: Vienna Ab-initio Simulation Package
XPS: X-ray photoelectron spectroscopy
XRD: X-ray diffraction
